# Drivers of students’ pro-environmental behavior in campus environments in Chinese university

**DOI:** 10.3389/fpsyg.2026.1760915

**Published:** 2026-02-16

**Authors:** Wen Chen, Sahar Erfanian

**Affiliations:** Business School, Huanggang Normal University, Huanggang, China

**Keywords:** biospheric value, connectedness to nature, environmental knowledge, green behavior, theory of planned behavior

## Abstract

**Introduction:**

Universities serve as vital platforms for shaping sustainability-oriented mindsets, particularly in rapidly transforming societies such as China. This study investigates the determinants of students’ pro-environmental behavior (PEB) within campus environments by employing an extended theory of planned behavior (ETPB) that integrates Connectedness to Nature (CN), Biospheric Values (BV), and Environmental Knowledge (EK) into the traditional framework.

**Methods:**

Using survey data from 431 students at a Chinese university, Structural Equation Modeling (SEM) was applied to examine the relationships among psychological, moral, and social predictors of PEB.

**Results:**

The extended model explained 63.8% of the variance in attitude and 78.6% of the variance in PEB, demonstrating strong explanatory power. Among traditional constructs, subjective norms exerted the strongest effect on PEB, followed by attitude and perceived behavioral control. CN, BV, and EK significantly influenced attitude but affected PEB indirectly, highlighting the mediating role of attitude in translating affective, cognitive, and moral antecedents into behavior.

**Discussion:**

The results underscore the social–moral nature of environmental engagement in collectivist contexts and emphasize the importance of integrating emotional, cognitive, and ethical education into sustainability initiatives. This study contributes theoretically to refining TPB for non-Western contexts and provides practical insights for advancing campus sustainability programs.

## 1 Introduction

Universities are crucial arenas for cultivating sustainability-oriented values and behaviors, particularly in societies undergoing rapid economic and environmental transformation (Fang et al., 2018; Maulana et al., 2025). As centers of knowledge creation and social development, universities have a profound influence on shaping the environmental consciousness of future leaders and professionals (Bibi et al., 2025). In China, the higher education sector occupies a pivotal position in advancing the national agenda for ecological civilization, a policy framework that integrates environmental protection into all aspects of development (Hou et al., 2025). The university campus, as a microcosm of society, provides both an educational and a social environment in which students can engage in and internalize sustainable or Pro-Environmental Behavior (PEB) (Chen et al., 2025; Maulana et al., 2025). Therefore, understanding the factors that shape PEB among university students is essential for promoting a culture of sustainability and supporting the broader environmental transition (Maleknia and Svobodova, 2025). PEB refers to actions individuals undertake to reduce environmental harm and contribute to ecological preservation. PEB includes behaviors such as recycling, energy conservation, sustainable consumption, and participation in green initiatives (Erfanian et al., 2024a).

A wide body of research has identified various psychological and sociocultural determinants of PEB, drawing on various theoretical frameworks (Al Mamun et al., 2022; AlHaddid et al., 2024; Maleknia and Enescu, 2025). These models such as Theory of Planned Behavior (TPB) emphasize how environmental attitudes, moral norms, and individuals’ perception of conducting behavior influence the likelihood of engaging in sustainable actions (Karimi and Mohammadimehr, 2022; Kotyza et al., 2024). The influence of these variables on PEB among young people and students has been confirmed in research (Karimi et al., 2021; Chen et al., 2025). However, PEB in different societies may be shaped by different determinants. The PEB is shaped by broader sociocultural contexts which influence operating primarily through socially embedded psychological mechanisms, particularly subjective norms and internalized environmental values such as Biospheric Values (BV) (Iwińska et al., 2023; Maleknia et al., 2024). In collectivist societies such as China, social expectations, institutional norms, and culturally grounded moral orientations are widely recognized as central pathways through which sociocultural factors shape individual behavior (Zhang M. et al., 2024; Qiu, 2025).

Chinese universities have made significant progress in implementing sustainability-oriented initiatives, such as green campus programs, environmental education courses, and waste classification systems (Winter et al., 2022; Zou et al., 2024). These institutional efforts are consistent with China’s national strategies which emphasize the integration of sustainability into university governance, teaching, and student life (Liu et al., 2024). Nonetheless, the translation of environmental awareness into consistent PEB among students remains uneven. Previous studies in suggest that while students often express strong environmental knowledge and concern, these factors do not always result in concrete behavioral change which might due to mediator factors such as attitude (AlHaddid et al., 2024; Tran et al., 2024). This outcome highlights the need to better understand the factors that might influence students’ attitude towards sustainable practices within campus settings.

Despite growing interest in sustainability education in China (Li et al., 2024; Liu et al., 2024; Zhang et al., 2025), existing research tends to focus on students’ environmental knowledge or attitudes rather than their actual behaviors and the contextual conditions influencing them (Fu et al., 2017; Xie and Lu, 2022). Furthermore, the literature has examined PEB from a psychological perspective (Wang and Zhang, 2021; Qiu, 2025). Although these studies highlighted the role of environmental knowledge (EK) or attitudes in PEB, they generally did not adopt models designed to examine the determinants of attitudes or to explore relationships beyond basic linear associations. Given the distinct sociopolitical and educational context of study area, a more comprehensive framework is required to capture the interplay between individual cognitions, social influences, and structural conditions. In fact, the study population in China is characterized by strong collectivist values, which may shape BV and context-specific subjective norms (Liu et al., 2024; Qiu, 2025). This approach can provide deeper insight into how campus environments either enable or constrain students’ PEB and how universities can act as transformative agents for sustainable development.

The present study therefore aims to investigate the determinants of PEB among university students. Specifically, the study aims to: (1) assess the relative effects of components of TPB on students’ PEB, (2) examine how connectedness to nature, biospheric values, and environmental knowledge shape PEB indirectly through attitude and directly. This study seeks to advance theoretical understanding of PEB in non-Western settings and provide empirical evidence to inform policy and practice in Chinese higher education by adopting an integrative and context-sensitive approach. The findings are expected to contribute to the development of more effective campus sustainability strategies and support China’s broader efforts toward building an ecologically responsible and sustainable society. This study aims to advance the literature on PEB behavior not by introducing new constructs, but by clarifying the functional roles and relative importance of established predictors within an integrated behavioral framework. By empirically testing an extended TPB in study university context, the study will provide insight into how affective, cognitive, and moral antecedents translate into behavior through distinct and uneven pathways.

### 1.1 Theoretical underpinning

This study employed an extended model of TPB to examine students’ behavioral patterns. The TPB, proposed by Ajzen (1985) has long served as one of the most influential models for predicting and explaining human behavioral intentions across a variety of domains, including environmentally responsible actions. The TPB posits that an individual’s behavioral intention defined as the motivational readiness to perform a particular action, is determined by three fundamental constructs. These constructs include attitude toward the specific behavior, subjective norm, and perceived behavioral control (Ajzen, 2011). Based on Ajzen (1993) attitude represents the individual’s overall evaluation of performing the behavior, whether favorable or unfavorable. Studies showed that environmental attitudes significantly increase the likelihood of adopting sustainable behaviors, including recycling, energy conservation, and green consumption (Liao et al., 2022; Duong, 2023). Within university settings, positive attitudes toward environmental protection are particularly salient, as students are frequently exposed to sustainability discourse through formal education and campus initiatives (Takshe et al., 2023). Subjective norm refers to the perceived expectations and social pressures from important referents such as peers, family, or institutions. Prior research consistently shows that social pressure plays a decisive role in shaping PEB (Morren and Grinstein, 2021; Savari and Khaleghi, 2023). This study hypothesizes that students’ environmental actions are strongly influenced by peer behavior, campus norms, and moral expectations embedded within higher education systems. Perceived behavioral control reflects an individual’s assessment of their capability and resources to perform the behavior (La Barbera and Ajzen, 2021). Studies have shown that higher perceived control enhances actual engagement in pro-environmental practices by reducing perceived barriers and increasing self-efficacy (Fawehinmi et al., 2024; Maleknia and Namdari, 2025).

Together, these factors predict behavioral intention, which subsequently leads to actual behavior. In the environmental domain, the TPB has been widely applied to predict behaviors such as energy conservation and recycling (Chen et al., 2024), sustainable consumption (Jebarajakirthy et al., 2024), and ecological activism (Rafiee et al., 2025), consistently demonstrating its robustness across cultural and contextual settings. In the original formulation of the TPB, behavioral intention is conceptualized as the immediate antecedent of action. However, in this study, intention is not included. In contexts where the research objective is to explain actual behavior rather than intention formation, studies have modeled behavior directly as a function of attitude, subjective norms, and PBC (Luh et al., 2023). This approach is particularly common in studies of PEB within structured or normative settings, such as university campuses, where behavioral opportunities and constraints are relatively well defined and actions may be habitual or socially regulated. In addition, prior research has noted that in cross-sectional survey designs, self-reported intention and self-reported behavior are often highly correlated, which can lead to conceptual redundancy and inflated path estimates (Savari and Khaleghi, 2023). Given these considerations, and in line with previous TPB-based environmental studies, the present research focuses on the direct determinants of PEB rather than modeling intention as a separate mediating construct. Hence, the foundational hypotheses of this study were formulated based on the role of the core components of the underlying model in shaping individual behavior. It was assumed that these three key components significantly influence the PEB of students. According to three basic components of original TPB, the following hypotheses were defined:

*H1*: The attitude of university students influences their PEB, positively.

*H2*: The perceived subjective norms of university students influence their PEB, positively.

*H3*: The university students’ perceived behavioral control influences their PEB, positively.

Nonetheless, the original TPB framework has been critiqued for its primarily rational-cognitive focus, which does not fully capture the affective, moral, and contextual dimensions of PEB (Sniehotta et al., 2014; St Quinton et al., 2021). Environmental decisions are often embedded in emotional connections, ethical considerations, and knowledge-based reasoning that extend beyond simple cost–benefit calculations (Kherazi et al., 2024; Lisboa et al., 2024). Consequently, researchers have developed various extensions of the TPB to enhance its explanatory power, particularly in sustainability studies where behavior is influenced by both internalized values and external conditions. The present study adopts an extended theory of planned behavior (ETPB) framework that incorporates three additional constructs including Connectedness to Nature (CN), BV, and EK into the traditional TPB model. These variables are included to capture the affective, moral, and cognitive mechanisms underlying students’ environmental engagement. Various studies extend the TPB by adding new predictors (Xing et al., 2023; Khan et al., 2024; Tan et al., 2024). Although these extended versions resulted in higher power of model prediction, but such extensions are often criticized for being linear or insufficiently theorized. To avoid an *ad hoc* expansion, the present study adopts a theoretically selective extension by integrating CN, BV, and EK, each representing a distinct motivational domain insufficiently captured by the original TPB. Together, CN, BV, and EK were selected to integrate affective, moral, and cognitive pathways into TPB, enhancing its explanatory depth while maintaining theoretical parsimony.

Although the new added constructs probably are empirically correlated with attitudes, they are conceptually distinct from the evaluative attitude construct within the TPB. According to Ajzen (2005) in TPB, attitude refers to an individual’s overall positive or negative evaluation of performing a specific behavior. By contrast, CN represents an affective and experiential bond with the natural world that reflects emotional identification rather than behavioral evaluation (Madera et al., 2024). EK constitutes a cognitive resource, capturing individuals’ awareness and understanding of environmental issues and response options, rather than their judgment of whether a given behavior is desirable (Nian et al., 2023). BV, in turn, reflect a stable normative orientation that prioritizes the intrinsic worth of nature and moral responsibility for its protection, operating at a deeper ethical level than situational evaluations (Stern et al., 1999). Conceptually, CN, EK, and BV function as upstream psychological dispositions that shape how individuals form evaluative judgments, rather than serving as evaluations themselves. Thus, while these constructs contribute to more favorable attitudes, they do not substitute for attitude within the TPB framework. From a theoretical perspective, modeling CN, BV, and EK as antecedents of attitude is consistent with hierarchical models of PEB, which posit that affective identification, moral values, and cognitive understanding serve as upstream determinants of evaluative appraisals. Empirically, although strong correlations are observed, discriminant validity tests confirm that these constructs are not interchangeable. Their indirect effects through attitude further support the conceptualization of CN, BV, and EK as formative influences on attitudes rather than as components of the attitude construct itself. This integrative ETPB framework moves beyond a purely rational–intentional model and provides a more comprehensive explanation of PEB in higher education and collectivist contexts.

CN describes the extent to which individuals perceive themselves as part of the natural world and experience an emotional, experiential, and spiritual bond with it (Mayer et al., 2009). Conceptually, it reflects an individual’s sense of inclusion within nature rather than separation from it. Individuals who feel a strong connection with nature are more likely to internalize environmental protection as part of their identity and thus to engage in behaviors that sustain ecological balance (Deville et al., 2021). A growing body of research indicates that CN is a significant predictor of environmental attitudes and behavioral intentions, and actual behavior (Korpilo et al., 2024; Krettenauer et al., 2024). Within the Chinese cultural setting, where traditional philosophies emphasize unity, balance, and respect for nature, the emotional and moral significance of CN may be particularly salient (Zhang et al., 2021). CN reflects individuals’ affective and experiential bonds with the natural environment, which precede rational evaluation and shape environmental attitudes through emotional identification. Unlike ecological identity, which emphasizes self-concept and social labeling, CN captures direct emotional engagement with nature and is particularly relevant in campus settings characterized by frequent everyday interaction with green spaces. Based on this theoretical foundation, these hypotheses were defined:

*H4*: The university students’ CN influences their attitudesو positively.

*H5*: The university students’ CN influences their PEB, positively.

BV represent enduring beliefs that prioritize the protection of the natural environment for its intrinsic worth rather than for utilitarian or egoistic reasons (Hu et al., 2024). These values are grounded in an ecological worldview that emphasizes the interdependence of all life forms (Kang et al., 2024). Empirical research grounded in the value-belief-norm theory (Stern et al., 1999) has shown that individuals with strong BV are more likely to develop personal norms and moral obligations toward sustainable behavior (Momenpour et al., 2024). By integrating BV into the TPB framework, the extended model captures the moral and altruistic motivations. This integration underline students’ environmental decisions, dimensions that are often underrepresented in the original rational-choice structure of TPB. In collective societies which welfare and moral education are key elements of socialization, BV can reinforce both individual and communal norms that support environmental protection (Wang et al., 2021). BV were incorporated to capture the moral foundations of PEB at a value level. While personal norms are often included in environmental models (Taso et al., 2020; Ambo Radde et al., 2024), they operate at a more proximal level and may conceptually overlap with subjective norms, especially in collectivist contexts. In contrast, BV represent deeper, more stable ethical orientations toward the intrinsic worth of nature, allowing moral motivation to be modeled without redundancy. Thus, BV was hypothesized to strengthen environmental attitudes toward PEB and also directly influence PEB:

*H6*: The university students’ BV influences their attitudes, positively.

*H7*: The university students’ BV influences their PEB, positively.

EK refers to an individual’s awareness and understanding of environmental issues, the functioning of natural systems, and the consequences of human actions on the environment (Kherazi et al., 2024; Sarwar et al., 2025a). Knowledge serves as the cognitive foundation for informed decision-making and effective behavior (Talha et al., 2025). A higher level of EK enhances individuals’ ability to recognize environmental problems, evaluate possible solutions, and assess their capacity to contribute to sustainability goals (Galván-Mendoza et al., 2022). Previous research suggests that knowledge not only shapes positive environmental attitudes but also increases perceived behavioral control by fostering confidence and perceived competence to engage in sustainable actions (Qayyoum and Hameed, 2023). In the Chinese university context, the integration of sustainability topics into curricula, national environmental education campaigns, and campus-wide initiatives probably plays an increasing role in strengthening students’ environmental literacy. The relationship between knowledge, attitude, and behavior in the KAP behavioral model has been confirmed in various studies (AlHaddid et al., 2024; Maleknia and Zamani, 2025), highlighting its influence through shaping attitudes. EK represents the cognitive foundation of informed environmental action. Unlike general environmental concern, which is conceptually close to attitude and risks constructs, EK captures individuals’ awareness and understanding of environmental issues and feasible responses. As such, EK functions as an upstream determinant of attitude rather than a parallel attitudinal construct. However, in this study, in addition to examining its indirect effect through attitudes, the direct impact of knowledge on behavior was also investigated to gain a more comprehensive understanding of how knowledge influences behavior. Accordingly, the following hypotheses were defined:

*H8*: The university students’ EK influences their attitudes, positively.

*H9*: The university students’ EK influences their PEB, positively.

**Figure 1:**
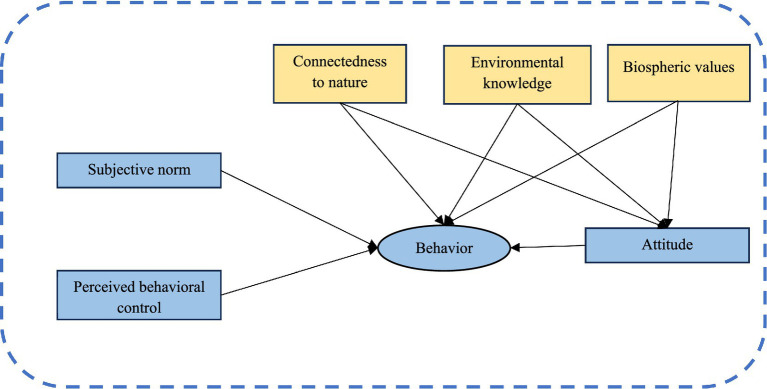
Theoretical framework of research.

The theoretical framework of the study is presented graphically in Figure 1.

## 2 Materials and methods

### 2.1 Study setting

This study was carried out in Huanggang, a prefecture-level city located in eastern Hubei Province, central China. As a significant cultural, educational, and ecological center, Huanggang spans both urban and rural landscapes and supports a population of approximately 6.3 million people. The city possesses an average of 12 square meters of urban green space per capita, reflecting local efforts to promote environmental livability and urban sustainability. Despite these initiatives, Huanggang faces several pressing environmental challenges, including soil erosion, seasonal waterlogging, and the fragmentation of urban green belts due to rapid urbanization. These issues underscore the necessity for sustainable urban planning and community engagement in environmental stewardship. Consequently, fostering environmentally responsible behavior among local residents is vital for the long-term protection and management of the city’s ecological resources. A key component of Huanggang’s social and cultural fabric is Huanggang Normal University, the only comprehensive institution of higher education in the region. Established in 1905, the university has a history of over a century and currently enrolls more than 20,000 students. It is recognized for its strong programs in teacher education, humanities, and applied sciences. As a major hub of intellectual and social development, the university plays a critical role in cultivating local human capital, advancing sustainability education, and promoting regional innovation. Its campus environment and academic community provide an ideal setting for examining sustainability-related attitudes and behaviors among university students within the broader context of China’s ecological civilization agenda.

### 2.2 Study participants and sampling method

Given the study’s focus on understanding PEB within the higher education context, students enrolled at Huanggang Normal University were selected as the target population. University students represent an ideal group for examining sustainability-related attitudes and behaviors because they are at a formative stage in developing environmental awareness and behavioral norms They are also directly influenced by institutional sustainability policies and campus culture. Moreover, as future educators, professionals, and community leaders, university students’ environmental values and actions have broader implications for promoting sustainable development within society. According to the Krejcie and Morgan (1970), a sample size of 384 is required for this population. However, to ensure greater accuracy, a larger sample size was utilized. The total number of participants in the study, after final data collection, reached 431, which is a substantial number for a study of this nature.

Data were collected using a structured online questionnaire survey, administered through secure digital platforms to ensure both efficiency and participant safety. The online survey method was particularly appropriate for this study for several reasons. First, it enabled broad and inclusive coverage of students across diverse academic disciplines, study levels, and demographic backgrounds, ensuring representation from multiple faculties within the university. Second, the online mode facilitated greater accessibility and convenience, allowing participants to complete the survey at their own pace and from any location with internet access. This method, thereby increases the likelihood of obtaining reliable and thoughtful responses. Third, this approach ensured anonymity and confidentiality, reducing the potential for social desirability bias and encouraging honest self-reporting of environmental attitudes and behaviors. From a methodological standpoint, online data collection also supports cost-effectiveness and logistical efficiency, enabling systematic and timely acquisition of a large volume of quantitative data suitable for statistical analysis. Additionally, considering the post-pandemic emphasis on minimizing physical contact and adhering to public health guidelines, the online format represented an ethical and pragmatic choice consistent with contemporary research standards in social science.

### 2.3 Data of study

The research data were collected through a questionnaire with items related to each of the model’s variables. These questions were designed based on the characteristics of the original model and subsequently adapted to fit the local context (Table 1). All measurement items were adapted from previously validated scales and modified to suit the university campus context. The original English items were translated into Chinese following a standard back-translation procedure to ensure semantic equivalence. First, the items were translated into Chinese by a bilingual researcher with expertise in environmental psychology. A second independent bilingual expert then back-translated the items into English. Discrepancies were discussed and resolved to ensure conceptual consistency with the original constructs. To enhance cultural and contextual relevance, items were further adapted to reflect students’ daily experiences within the university environment, particularly for constructs such as CN and BV, which may carry culturally specific interpretations. The PBE was measured as a general behavioral construct to capture students’ overall tendency toward environmentally responsible conduct in the campus context, where sustainability engagement typically involves multiple, interrelated practices. This approach aligns with the study’s focus on how broad psychological antecedents translate into generalized behavioral engagement rather than isolated actions. Before the final use of the questionnaire, it underwent two evaluation phases. Initially, a six-member team of specialists in behavioral science, environmental studies, and social sciences reviewed the questions to assess their validity. Based on the feedback from this team, the questionnaire was revised. In the subsequent phase, the questionnaire was tested in a pre-test involving 30 participants, achieving a Cronbach’s alpha of over 0.7, which confirmed its reliability (Cronbach, 1951). Following this two-step validation process, the questionnaire was employed for the final data collection phase. The first page of the questionnaire provided details about the research objectives to the students. They were assured that no personal information would be collected. Before completing the questionnaire, participants indicated their consent to participate in the study by checking a box on the final page.

**Table 1:** The constructs of research model with their statements.

Construct	Code	Statement	Factor loading	Origin
Attitude	ATT1	Protecting the university environment is important to me.	0.91	Li et al. (2023); Wang et al. (2024)
	ATT2	I feel positive about initiatives on campus that promote environmental protection.	0.93	
	ATT3	Supporting environmental conservation is a wise and necessary action for students.	0.91	
	ATT4	I value the preservation of natural areas and green spaces within and around the university.	0.89	
Environmental knowledge	EK1	I am well informed about environmental issues that affect the university and society.	0.87	Zeng et al. (2023); Thuy et al. (2024)
	EK2	I know what actions I can take as a student to help protect the environment.	0.91	
	EK3	I understand how environmental conservation contributes to the well-being of students and society.	0.83	
Connectedness to nature	CON1	I feel emotionally attached to the natural environment around the university.	0.84	Barrera-Hernández et al. (2020); Douglas et al. (2024)
	CON2	Spending time in nature or green spaces on campus makes me feel connected to the environment.	0.87	
	CON3	I would be upset if the natural environment on or near campus were damaged or destroyed.	0.86	
	CON4	I feel a sense of belonging when I spend time in natural settings on or near the university.	0.87	
Biospheric values	BV1	I believe protecting nature is important even if it does not directly benefit people.	0.65	Zhang et al. (2020); Maleknia et al. (2024)
	BV2	I think all living things have a right to exist.	0.75	
	BV3	I care deeply about damage done to the natural environment.	0.88	
	BV4	I feel personally responsible for minimizing harm to nature.	0.81	
Subjective norms	SN1	People important to me think I should engage in environmentally friendly behaviors.	0.90	Druica et al. (2023); Erfanian et al. (2024b)
	SN2	My friends and classmates expect me to care about environmental protection.	0.90	
	SN3	There is strong social support among students for protecting the environment.	0.91	
Perceived behavioral control	PBC1	I feel capable of taking actions that help protect the environment as a student.	0.89	Druica et al. (2023); Zhang M. et al. (2024)
	PBC2	It would be easy for me to participate in environmental activities organized by the university.	0.89	
	PBC3	I can find opportunities on or off campus to support environmental conservation if I want to.	0.90	
Pro-environmental behavior	PB1	I often engage in environmentally responsible behaviors in my daily student life.	0.88	
	PB2	I have participated in campus or community activities that promote environmental protection.	0.88	
	PB3	I usually behave in ways that help keep the campus and surrounding environment clean and healthy.	0.88	

### 2.4 Data analysis

The data were analyzed using Structural Equation Modeling (SEM) to examine the measurement and structural relationships among the study constructs. The analysis followed a two-step approach. First, assessing the measurement model for reliability and validity, and then testing the structural model to evaluate the hypothesized relationships.

#### 2.4.1 Reliability and validity

To evaluate the reliability of each construct, Cronbach’s alpha (*α*) and Composite Reliability (CR) values were computed. Both indicators assess internal consistency, with thresholds of 0.70 or higher considered acceptable (Hair et al., 2017). Convergent validity was examined through the Average Variance Extracted (AVE), which represents the proportion of variance explained by the construct relative to measurement error. An AVE value exceeding 0.50 indicates adequate convergent validity. Discriminant validity was assessed using the Fornell and Larcker (1981) criterion. According to this method, the square root of each construct’s AVE should exceed the correlations between that construct and any other construct in the model. This procedure was applied to verify that all latent constructs are conceptually distinct and that the observed indicators measure unique dimensions of the theoretical framework. Given that the data were collected from a single source using a self-administered questionnaire, the potential influence of common method bias was examined. Following established recommendations, Harman’s single-factor test was employed as a diagnostic procedure (Al Mamun et al., 2022). All measurement items were entered into an unrotated exploratory factor analysis to determine whether a single latent factor accounted for the majority of the covariance among the measures. The results indicated that the first factor explained less than 50% of the total variance, suggesting that common method variance is unlikely to be a serious concern in this study.

#### 2.4.2 Structural model and hypothesis testing

After confirming the adequacy of the measurement model, the structural model was tested to evaluate the hypothesized causal relationships among constructs. Path coefficients (*β*), coefficient of determination (*R*^2^), and significance levels were estimated using a bootstrapping procedure with 5,000 resamples. The significance of each hypothesized relationship was determined based on the *t*-statistics and *p*-values derived from the bootstrapped estimates.

## 3 Results

### 3.1 Results of reliability and validity

Table 2 presents the results of the measurement model assessment in terms of internal consistency reliability and convergent validity. All constructs demonstrated satisfactory reliability, with Cronbach’s alpha values exceeding the recommended threshold of 0.70 (Cronbach, 1951). Specifically, Cronbach’s alpha coefficients ranged from 0.78 for BV to 0.931 for attitude, indicating strong internal consistency among the items. Similarly, CR values were all above 0.85, ranging between 0.859 and 0.951, confirming the reliability and internal consistency of the constructs (Hair et al., 2017). Moreover, the AVE values were well above the recommended threshold of 0.50, thereby establishing satisfactory convergent validity. These results collectively indicate that all latent constructs in the model exhibit acceptable reliability and validity and are therefore suitable for further structural analysis. Table 3 presents the Fornell–Larcker criterion results used to assess discriminant validity among the study constructs. As shown in the table, the diagonal values range from 0.779 to 0.903, all of which exceed the corresponding inter-construct correlations in their respective rows and columns. This pattern indicates that each construct shares more variance with its own indicators than with other constructs, thereby confirming adequate discriminant validity (Fornell and Larcker, 1981). These findings demonstrate that the constructs in the model are empirically distinct and that the measurement model satisfies the required validity conditions for subsequent structural analysis. The factor loadings of all measurement items are illustrated in Table 1. All loadings exceed the recommended threshold (Hair et al., 2017), further confirming the reliability and convergent validity of the constructs.

**Table 2:** The reliability and validity of research model.

Constructs	Cronbach’s alpha	CR	AVE
Attitude	0.931	0.951	0.828
Biospheric values	0.780	0.859	0.606
Connectedness to nature	0.880	0.918	0.736
Environmental knowledge	0.836	0.902	0.755
Perceived behavioral control	0.872	0.921	0.796
Pro-environmental behavior	0.852	0.910	0.772
Subjective norms	0.887	0.930	0.816

**Table 3:** The discriminant validity of model.

Constructs	1	2	3	4	5	6	7
Attitude	0.910						
Biospheric values	0.766	0.779					
Connectedness to nature	0.687	0.741	0.858				
Environmental knowledge	0.651	0.640	0.724	0.869			
Perceived behavioral control	0.739	0.788	0.664	0.614	0.892		
Pro-environmental behavior	0.797	0.754	0.672	0.691	0.763	0.878	
Subjective norms	0.736	0.698	0.604	0.716	0.744	0.829	0.903

### 3.2 Structural model results

**Figure 2:**
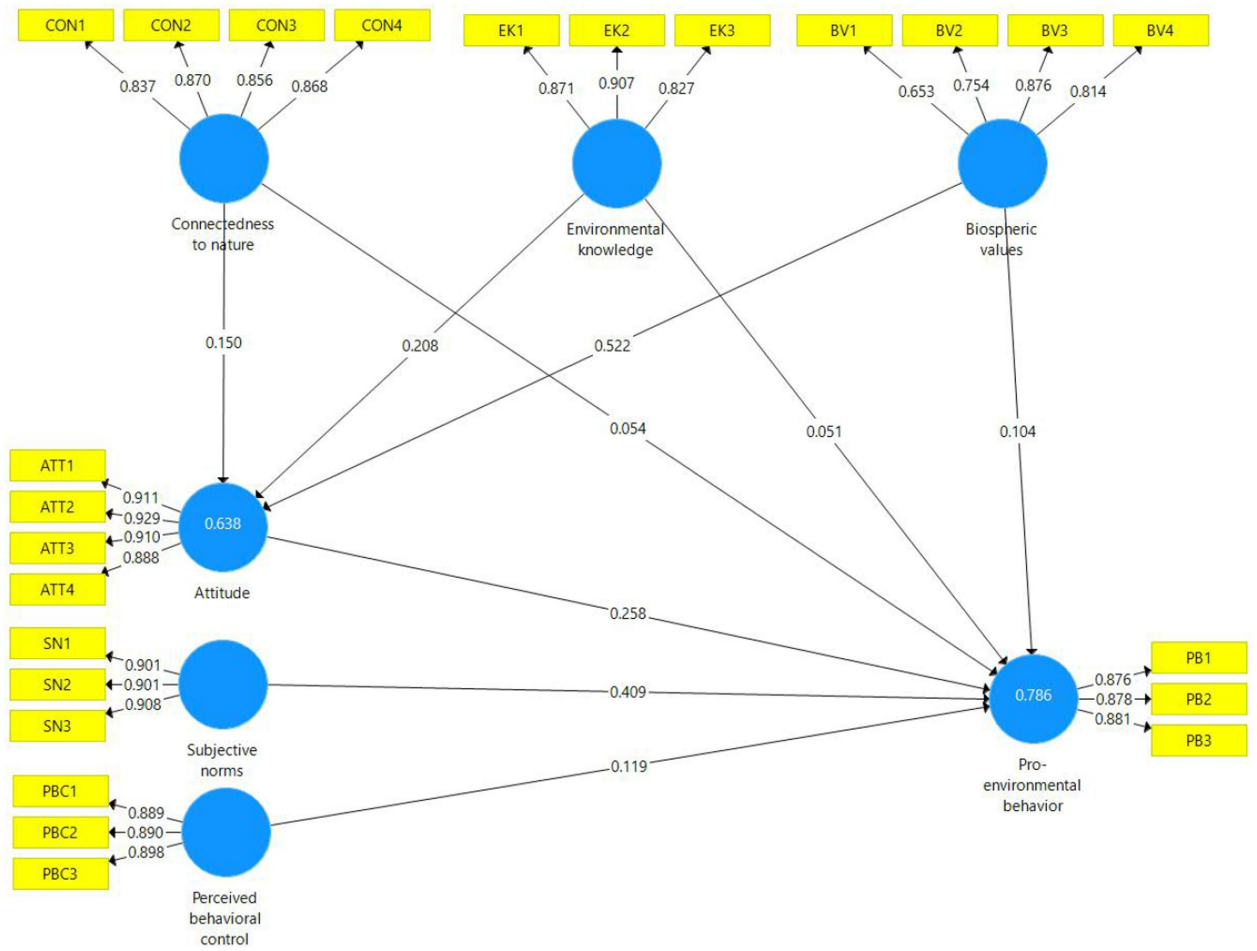
The structural model of research.

The structural model was estimated to assess the hypothesized relationships among the constructs of the ETPB. The model (Figure 2) demonstrated strong explanatory power, accounting for 63.8% of the variance in attitude and 78.6% of the variance in PEB. Among the traditional TPB variables, subjective norms (*β* = 0.409, *t* = 7.598, *p* < 0.001) had the strongest direct effect on PEB, followed by attitude (*β* = 0.258, *t* = 4.092, *p* < 0.001) and perceived behavioral control (*β* = 0.119, *t* = 2.059, *p* = 0.040). These results indicate that all three TPB constructs significantly contributed to the prediction of PEB, with subjective norms exerting the greatest influence.

Regarding the extended variables, CN significantly affected attitude (*β* = 0.150, *t* = 1.975, *p* = 0.049) but did not show a significant direct effect on PEB (*β* = 0.054, *t* = 0.783, *p* = 0.434). EK also showed a significant path to attitude (*β* = 0.208, *t* = 3.891, *p* < 0.001) but not to PEB (*β* = 0.051, *t* = 1.006, *p* = 0.315). BV demonstrated a strong and significant effect on attitude (*β* = 0.522, *t* = 7.991, *p* < 0.001) but a non-significant direct relationship with PEB (*β* = 0.104, *t* = 1.716, *p* = 0.087). The analysis of indirect and total effects showed that CN (total effect = 0.093), EK (total effect = 0.105), and BV (total effect = 0.239) influenced PEB mainly through attitude (Table 4). This indicates that these antecedent constructs contribute to PEB indirectly rather than through direct paths. Overall, six out of nine hypotheses (H1, H3, H5, H7, H8, and H9) were supported (Table 5). The results confirm that attitude, subjective norms, and perceived behavioral control significantly predict PEB, while CN, EK, and BV exert significant effects on attitude, which in turn serves as an important pathway linking these antecedents to PEB.

**Table 4:** The direct, indirect and total effects of constructs.

Variables	Direct effect	Indirect effect	Total effect
Attitude	0.258		0.258
Subjective norms	0.409		0.409
Perceived behavioral control	0.119		0.119
Connectedness to nature	0.054	0.039	0.093
Environmental knowledge	0.051	0.054	0.105
Biospheric values	0.104	0.134	0.239

**Table 5:** The results of hypothesis test of research.

Hypotheses		*t* value	*p* values	Result
H1	Connectedness to nature > attitude	1.975	0.04	Confirmed
H2	Connectedness to nature > pro-environmental behavior	0.783	0.43	Rejected
H3	Environmental knowledge > attitude	3.891	0.00	Confirmed
H4	Environmental knowledge > pro-environmental behavior	1.006	0.31	Rejected
H5	Biospheric values->attitude	7.991	0.00	Confirmed
H6	Biospheric values > pro-environmental behavior	1.716	0.08	Rejected
H7	Attitude > pro-environmental behavior	4.092	0.00	Confirmed
H8	Subjective norms > pro-environmental behavior	7.598	0.00	Confirmed
H9	Perceived behavioral control > pro-environmental behavior	2.059	0.04	Confirmed

## 4 Discussion

The present study examined the determinants of PEB among university students in China by extending the TPB to include three antecedent constructs including CN, EK, and BV. The extended model demonstrated substantial explanatory power, accounting for 63.8% of the variance in attitude and 78.6% of the variance in PEB, which reflects a robust capacity to explain the psychological and contextual factors underlying students’ sustainability-related actions. These findings reinforce the view that PEB among young people is a multifaceted phenomenon shaped by affective, cognitive, and moral dimensions, in addition to traditional social-cognitive predictors.

### 4.1 Effects of the traditional TPB constructs

The results confirmed the predictive strength of the original TPB components in explaining students’ engagement in environmentally responsible behavior. Attitude exerted a significant and positive influence on PEB (H1), confirming that favorable evaluations of environmental protection enhance the likelihood of engaging in sustainable actions. This outcome aligns with the core assumptions of TPB and is widely supported in environmental psychology literature (Ajzen, 1991; Li et al., 2023). Numerous studies have shown that individuals with stronger pro-environmental attitudes are more likely to participate in behaviors such as recycling, energy conservation, and sustainable consumption (Jebarajakirthy et al., 2024; Tran et al., 2024). The results of the current study reinforce this pattern within the context of Chinese university students, indicating that positive emotional and cognitive appraisals of environmental actions remain central determinants of behavior even when extended variables are incorporated. This finding indicates that strengthening the manifestation of PEB among students requires the enhancement of their environmental attitudes. Accordingly, educational programs aimed at fostering such attitudes can be promoted and reinforced as an effective strategy within the university context.

The subjective norms exhibited the most substantial direct effect on PEB, suggesting that perceived expectations from peers, family, and university authorities are powerful motivators for students’ environmental actions. This finding is consistent with research emphasizing the dominant role of social influence in collectivist cultures such as China, where conformity and interdependence often outweigh individual attitudes in shaping behavioral choices (Maulana et al., 2025). Within university settings, the influence of classmates, student organizations, and campus environmental campaigns (Sarwar et al., 2025b), likely reinforces the perception that sustainable behavior is both socially approved and morally expected. The strong predictive power of subjective norms also reflects the institutional structure of Chinese higher education, where moral education and civic responsibility are integrated into curricula and student life. Prior studies have reported similar patterns in Asian and Middle Eastern contexts, indicating that PEB among students is frequently guided by collective expectations and perceived social obligations rather than purely individual preferences (Bibi et al., 2025). These findings suggest that interventions aimed at promoting sustainability in higher education may achieve greater effectiveness when they leverage peer influence, social recognition, and institutional norms rather than focusing solely on personal motivation.

PBC also significantly predicted PEB, though its effect was smaller than that of attitude and subjective norms. This suggests that while students’ perceived capability to act pro-environmentally matters, behavioral performance is influenced more strongly by social pressure and attitudinal conviction. Similar findings have been reported by Krettenauer et al. (2020), who observed that PBC tends to play a limited role when external support and normative pressure are strong. The modest influence of PBC in this study could also be related to contextual barriers such as limited institutional resources or infrastructural constraints that restrict students’ opportunities to act, despite their willingness. Accordingly, it can be argued that planning is required in two key dimensions including enhancing individuals’ capacity to engage in PEB and providing the conditions that enable the expression of such behaviors. Therefore, educational interventions are necessary to strengthen individuals’ abilities and competencies to perform these behaviors; on the other hand, it is essential to assess and ensure the availability of the necessary infrastructural and resource-related conditions that facilitate their implementation.

### 4.2 Contributions of the extended variables

CN significantly influenced attitude but not PEB directly. This finding suggests that emotional attachment to the natural environment enhances students’ positive evaluations of sustainable behavior, which in turn leads to behavioral engagement through attitudinal mediation. This pattern is consistent with previous research showing that CN often exerts indirect rather than direct effects on PEB (Deville et al., 2021). CN encapsulates affective and experiential dimensions of environmental identity; individuals who perceive themselves as part of nature tend to internalize ecological values, forming stronger pro-environmental attitudes (Douglas et al., 2024). On university campuses, frequent exposure to natural spaces, environmental clubs, or outdoor educational programs may enhance CN, which subsequently strengthens attitudinal predispositions toward sustainability. Accordingly, the creation and expansion of natural spaces within the university environment, as well as the implementation of nature-based visitation programs, can strengthen this variable and, in turn, contribute to the enhancement of PEB.

EK also emerged as a significant antecedent of attitude but did not directly predict PEB. This supports the long-standing argument that knowledge is a necessary but insufficient condition for behavioral change (Qayyoum and Hameed, 2023). Knowledge enhances understanding and awareness of environmental problems, yet the translation of awareness into behavior requires motivational and normative reinforcement. The insignificant direct effect of EK on behavior is consistent with the knowledge–action gap frequently reported in environmental studies (Yu and Zhu, 2016; St Quinton et al., 2021). Nonetheless, its significant indirect impact through attitude demonstrates that informed individuals are more likely to develop favorable perceptions of sustainability, which serve as cognitive foundations for later behavioral engagement (Liao et al., 2022). Within Chinese higher education, where environmental topics are increasingly integrated into general curricula, this finding implies that the educational system effectively cultivates awareness but may need to place stronger emphasis on experiential learning and participatory activities that convert awareness into action.

BV showed the strongest positive effect on attitude and the highest total effect on PEB among the antecedent constructs. This indicates that value-based orientations are fundamental in shaping students’ environmental commitment. The result resonates with findings from value-belief-norm theory, which asserts that biospheric values motivate individuals to act out of moral obligation to protect nature (Stern et al., 1999; Adjei et al., 2023). Although BV did not directly predict behavior, its substantial indirect influence via attitude highlights the mediating role of cognitive appraisal in transforming moral concern into concrete actions (Maleknia et al., 2024). The prominence of BV in this study reflects the integration of moral education within Chinese universities, where collective well-being and ecological responsibility are emphasized as ethical imperatives. This moral framing of sustainability distinguishes the Chinese context from many Western studies, where PEB is often interpreted through individualistic frameworks emphasizing self-efficacy and personal benefit. Accordingly, raising awareness of BV among university students can indirectly contribute to the strengthening of pro-environmental behavior. This finding clearly highlights the importance of environmental education and the reinforcement of BV.

The finding that CN, EK, and BV primarily influence PEB through attitude underscores the central role of evaluative processing in students’ behavior formation. For university students, emotional attachment to nature, moral concern for the biosphere, and EK function as latent motivational resources that do not automatically translate into action. Instead, these factors require integration into favorable behavioral evaluations, indicating that attitude serves as a psychological bridge through which affective, moral, and cognitive inputs are transformed into behavioral readiness (Qayyoum and Hameed, 2023). Within higher education contexts, EK and values are often acquired in abstract or normative forms, while opportunities for immediate action may be constrained by institutional routines and social expectations. Under such conditions, CN, EK, and BV first strengthen positive evaluations of PEB, which then increase the likelihood of action when situational opportunities arise. This pattern suggests that student PEB is largely deliberative rather than automatic. From a contrasting perspective, CN, EK, and BV can exert direct effects on behavior when environmental identity is strongly internalized, behaviors are habitual, or institutional infrastructures actively support sustainable action (Maleknia et al., 2025). In such contexts, moral or affective motivations may bypass attitudinal deliberation and directly guide behavior.

This result clearly demonstrates the role of these variables in shaping and strengthening attitudes. Accordingly, the findings indicate that individuals’ attitudes can be formed under the influence of affective, cognitive, and knowledge-based factors. To strengthen attitudes which constitute the core component of the traditional TPB model, it is necessary to reinforce these underlying components. These results therefore provide evidence for the robustness of the proposed model in effectively incorporating these variables. The absence of strong direct effects in this study therefore reflects the developmental and contextual nature of student behavior rather than the limited importance of these constructs. Overall, the findings imply that attitude formation is a critical mechanism for translating internal dispositions into PEB in university settings.

The non-significant direct effects of CN, EK, and BV on PEB suggest that these constructs operate primarily as distal antecedents rather than immediate behavioral drivers (Maleknia et al., 2024). Although prior studies report mixed results, the present findings indicate that emotional attachment to nature, environmental knowledge, and biospheric values influence behavior indirectly by shaping favorable environmental attitudes. This pattern aligns with extended TPB assumptions, which posit that affective, cognitive, and moral dispositions must first be cognitively evaluated before translating into action. In the study area context, where social expectations and collective norms play a dominant role, individual-level dispositions may remain behaviorally latent unless reinforced by positive attitudes and normative support. The strong effect of subjective norms observed in this study supports this interpretation.

### 4.3 Integrated interpretation of the extended model

The findings collectively demonstrate that attitude serves as the central mechanism linking affective, cognitive, and moral antecedents to PEB. This mediating function underscores the multidimensional nature of behavioral formation. The emotional connection to nature, knowledge-based understanding, and moral valuation of the biosphere converge to generate favorable attitudes, which subsequently lead to behavioral engagement. This result aligns with integrative behavioral models that emphasize the dynamic interplay between cognition, emotion, and moral judgment in sustainability decision-making. The strong role of subjective norms, combined with the indirect pathways from CN, EK, and BV through attitude, reveals a distinctive pattern of social–moral environmental engagement among Chinese university students. Behavior appear to emerge not only from personal evaluation but also from collective reinforcement and moral legitimacy. In this regard, the ETPB effectively captures both individual and contextual determinants of PEB in non-Western educational settings, offering a culturally sensitive framework for understanding sustainability engagement in collectivist societies. Overall, the results reveal that sustainable behavior among university students is not solely a rational process but emerges from the interaction of social pressure, emotional identification with nature, cognitive awareness, and moral conviction. These findings highlight the need for multidimensional strategies in environmental education that simultaneously cultivate knowledge, strengthen affective bonds with nature, and reinforce social and moral norms supportive of sustainability. By empirically validating this expanded theoretical model, the study contributes to ongoing efforts to refine behavioral theories of sustainability and offers a conceptual bridge between Western psychological models and the moral–collectivist orientation characteristic of East Asian cultural contexts.

Beyond individual psychological determinants, the findings should be interpreted in light of the broader contextual and institutional environment in which students’ PEB is embedded. University infrastructure and policy frameworks shape the extent to which favorable attitudes, social norms, and perceived control can be translated into actual behavior. For example, campus facilities such as waste separation systems, energy-efficient buildings, accessible green spaces, and organized environmental programs provide concrete opportunities for action, thereby strengthening the behavioral relevance of perceived behavioral control (Maulana et al., 2025). Conversely, limited infrastructure may constrain behavior even among highly motivated students. Institutional policies including formal sustainability strategies, compulsory environmental courses, and administrative endorsement of green initiatives also play a critical role by legitimizing PEB and reinforcing subjective norms. In the Chinese higher education context, where institutional authority and policy guidance are particularly influential, such structural conditions may amplify the effect of social norms while simultaneously reducing the relative importance of individual agency (Zou et al., 2024). This contextual embedding helps explain why subjective norms emerged as the strongest predictor of behavior in this study and why environmental knowledge and values primarily operated through attitudinal pathways rather than exerting direct behavioral effects. Thus, students’ PEB should be understood as the outcome of an interaction between psychological predispositions and enabling or constraining institutional environments.

## 5 Implications

### 5.1 Practical implications

The findings of this research have practical implications which should be interpreted specifically within the context of Chinese higher education. In this context, PEB is shaped by collectivist cultural norms, strong institutional guidance, and state-led sustainability agendas. First, the dominance of subjective norms observed in this study reflects the central role of social expectations, peer influence, and institutional authority in shaping student behavior in China, rather than universal behavioral mechanisms. Second, the strong effect of subjective norms suggests that Chinese universities can most effectively promote students’ PEB by reinforcing collective expectations and visible social endorsement of sustainability practices. Campus-based initiatives such as student party organizations, Youth League activities, dormitory-based competitions, and public recognition of environmentally responsible groups are particularly well aligned with the Chinese educational environment, where behavior is strongly influenced by peer conformity and institutional signaling. Third, the mediating role of attitude indicates that sustainability education should move beyond information transmission and actively cultivate positive evaluative orientations toward environmental protection. Integrating sustainability themes into ideological and moral education courses, general education curricula, and campus cultural activities can help embed environmental responsibility within students’ broader value systems, which is consistent with China’s emphasis on moral cultivation and collective responsibility.

Fourth, the indirect effects of BV and CN highlight the importance of moral and affective pathways that resonate with traditional Chinese ecological philosophies emphasizing harmony between humans and nature. Universities can strengthen these pathways by incorporating nature-based learning, campus green space engagement, and reflective activities that connect environmental protection with ethical citizenship and social responsibility. Finally, the limited direct effect of EK underscores a context-specific knowledge–action gap within the higher education. While EK is increasingly institutionalized through national curricula and policy directives, translating knowledge into behavior requires strong normative reinforcement and structured opportunities for action. Universities should therefore focus on practice-oriented programs such as mandatory participation in sustainability projects or institutionally organized environmental campaigns that align individual behavior with collective goals. Overall, these implications are not intended to be generalized across cultural contexts. Rather, they reflect the specific sociocultural and institutional conditions of case study in China, where PEB is primarily driven by social legitimacy, moral education, and collective norms rather than individual autonomy alone.

### 5.2 Theoretical implications

This study contributes to the theoretical advancement of PEB research by refining and extending the TPB within a higher education and non-Western context. By integrating connectedness to nature, EK, and BV into the TPB framework, the study responds directly to long-standing critiques of TPB’s predominantly rational–cognitive orientation. The findings demonstrate that incorporating affective and moral antecedents enhances the model’s explanatory capacity, thereby supporting a more comprehensive representation of environmental decision-making processes. A key theoretical contribution lies in clarifying the structural role of attitude as a central mediating mechanism. The results show that affective (CN), cognitive (EK), and moral (BV) factors do not translate directly into behavior but operate through individuals’ evaluative judgments. This layered influence structure provides empirical support for a hierarchical pathway in which upstream psychological dispositions shape behavior indirectly via attitude formation. By formally validating these indirect pathways within a structural equation modeling framework, the study advances understanding of how multiple psychological domains interact in the formation of PEB. A key insight of this study lies in demonstrating that the effectiveness of integrated behavioral models depends not only on which constructs are included, but on how their causal roles are specified. The dominance of subjective norms, combined with the indirect effects of values, knowledge, and CN, points to a social–moral pathway of PEB that differs from individualistic, control-based explanations commonly reported in Western contexts. This finding helps explain inconsistencies in prior studies and underscores the importance of contextualizing integrated models rather than assuming universal causal structures.

The integration of constructs derived from distinct theoretical traditions represents another important contribution. By synthesizing elements from TPB, value–belief–norm theory, and the connectedness to nature perspective, the extended model bridges previously fragmented strands of PEB research. Rather than treating rational evaluation, moral obligation, and emotional identification as competing explanations, the findings demonstrate their complementary roles within a unified explanatory structure. This integrative approach supports the view that PEB emerges from the convergence of cognitive appraisal, moral orientation, and affective connection, rather than from any single motivational source. Finally, the study contributes to the cross-cultural validation of behavioral theory by demonstrating the contextual sensitivity of TPB-based models. The findings underscore that the relative strength of TPB components is culturally contingent and that behavioral models developed in Western contexts require contextual adaptation when applied elsewhere. By empirically validating an extended TPB in a university setting, the study provides evidence for the flexibility of the framework and contributes to the development of culturally responsive theories of PEB.

## 6 Limitations and future research directions

While the present study makes significant theoretical and empirical contributions to understanding PEB among university students, several limitations should be acknowledged to guide cautious interpretation of the results and to inform future research. Recognizing these boundaries not only strengthens the credibility of the findings but also highlights promising avenues for continued theoretical refinement and empirical exploration. A primary limitation of this study lies in its cross-sectional research design, which captures relationships between constructs at a single point in time. Although structural equation modeling allows for the estimation of directional paths based on theory, causal inferences cannot be definitively established. The observed relationships particularly the mediating role of attitude and the indirect effects of CN, EK, and BV should therefore be interpreted as correlational rather than causal. Future research could employ longitudinal or experimental designs to examine how changes in these antecedent variables over time influence the formation and persistence of pro-environmental intentions and behaviors. Such designs would enable scholars to test causal mechanisms more robustly, evaluate the stability of attitudes and norms, and identify potential reciprocal relationships between behavior and psychological constructs. Despite its theoretical justification, the exclusion of behavioral intention represents a limitation of the present study. Intention may serve as a mediating mechanism through which attitudes and norms translate into action, particularly in less structured or more voluntary behavioral contexts. Future research should incorporate intention explicitly and employ longitudinal or experimental designs to better distinguish between intention formation and behavioral enactment.

A key limitation of this study is that data were collected from a single university, which constrains the external validity of the findings. While the university represents a typical comprehensive institution in central China, institutional culture, geographic location, and sustainability practices vary widely across Chinese higher education institutions. As a result, the findings should be understood as illustrative rather than nationally generalizable. Future research should employ multi-site sampling across universities with diverse academic orientations, governance structures, and regional contexts to assess the robustness and generalizability of the proposed model at the national level. Furthermore, university students represent a relatively homogeneous group in terms of age, education, and social exposure, which may not reflect the diversity of attitudes and constraints present in the general population. To enhance external validity, future studies should consider employing multi-site sampling across universities with different academic orientations, governance structures, and geographic regions. Comparative studies across countries or cultural contexts particularly between collectivist and individualist societies, would also help to identify universal versus culturally contingent components of the ETPB. Such cross-cultural validation would be essential for refining the model’s global applicability and testing its cultural sensitivity.

Another limitation concerns the reliance on self-reported data, which are inherently subject to social desirability and self-perception biases. Students may have overstated their environmentally responsible attitudes or behaviors in order to align with perceived social expectations, particularly given the increasing institutional emphasis on sustainability. Although anonymity and confidentiality were ensured, this limitation cannot be completely ruled out. Future studies could mitigate this concern by incorporating objective behavioral measures such as energy consumption monitoring, waste-sorting participation records, or digital tracking of eco-friendly actions on campus. Triangulating self-reports with observational or experimental data would provide a more accurate picture of actual behavioral patterns and help to validate self-reported constructs. The use of implicit attitude measures or behavioral experiments could also reveal subconscious influences and reduce self-presentation effects.

An additional limitation of this study concerns the exclusion of behavioral intention from the analytical model. Although this decision was theoretically justified and consistent with prior TPB-based studies focusing on actual behavior in structured settings, excluding intention may underestimate certain indirect motivational mechanisms through which attitudes, subjective norms, and perceived behavioral control influence behavior. In particular, intention often functions as an intermediate cognitive–motivational state that translates evaluative and normative influences into action, especially in contexts where behavior is less habitual or institutionally guided. As a result, the present model may not fully capture the sequential motivational pathways linking upstream psychological dispositions to pro-environmental behavior. Accordingly, the findings should be interpreted as reflecting the direct and attitudinally mediated determinants of behavior rather than the complete intention–behavior process. Future research should explicitly incorporate behavioral intention and examine its mediating role using longitudinal or experimental designs to provide a more comprehensive account of motivational dynamics.

Although the ETPB substantially increased the predictive power of the original TPB, the model does not exhaust all possible determinants of sustainable behavior. Several relevant constructs—such as environmental identity, ecological worldview, moral norms, and emotional empathy—may further illuminate the complex motivational structure underlying PEB. Moreover, contextual and structural factors, such as campus infrastructure, policy support, and availability of green resources, could moderate the strength of psychological determinants. Future research should therefore consider extending the model by incorporating multi-level and contextual variables, exploring interactions between individual-level cognitions and institutional or environmental enablers. Mixed-method approaches combining quantitative modeling with qualitative inquiry could also provide deeper insights into how students interpret and negotiate sustainability challenges within their lived experiences. This study does not explicitly model sociocultural differences across regions, institutions, or social groups. Although sociocultural influences are theoretically captured through subjective norms and value-based constructs, the absence of direct contextual indicators limits the ability to disentangle specific cultural or institutional effects. Future research should incorporate cross-university or cross-regional samples and apply multi-group or multilevel modeling approaches to more directly examine sociocultural heterogeneity in PEB. Finally, while this study acknowledges the importance of cultural context, further investigation is warranted into how traditional ecological philosophies, shape the moral and affective foundations of PEB. Understanding how these indigenous philosophical frameworks interact with modern behavioral models could offer valuable theoretical enrichment. Comparative studies could explore how spiritual or ethical worldviews contribute to the formation of biospheric values and connectedness to nature across different cultural paradigms.

## 7 Conclusion

This study set out to investigate the determinants of PEB among university students in China through an ETPB that integrates CN, EK, and BV into the traditional TPB framework. The results demonstrated that the extended model possesses strong explanatory power, accounting for over 60% of the variance in attitude and nearly 78% of the variance in actual PEB. These findings provide compelling evidence that sustainable behavior among university students is shaped by a complex interplay of emotional, social, cognitive, and moral factors. Among the traditional TPB variables, subjective norms emerged as the most influential predictor, underscoring the importance of social influence and collective expectations in shaping environmentally responsible behavior within collectivist educational environments. Attitude was also a robust predictor of PEB and served as a critical mediating variable through which CN, EK, and BV exerted indirect effects. PBC had a smaller yet significant influence, indicating that structural and self-efficacy factors play a supporting, though not dominant, role. The extended variables contributed substantially to explaining variations in environmental attitudes. CN fostered favorable environmental evaluations through affective identification with the natural world. EK enhanced cognitive understanding and awareness, thereby strengthening attitudinal commitment. BV represented the strongest moral antecedent, linking ethical concern for nature to positive environmental evaluations and indirect behavioral engagement. Together, these constructs capture the emotional, moral, and informational foundations of sustainability behavior among young adults.

Theoretically, this study advances behavioral science by demonstrating that the TPB can be effectively expanded to incorporate non-rational dimensions of environmental engagement. The integration of CN, EK, and BV not only addresses long-standing critiques of TPB’s cognitive focus but also bridges it with affective and moral frameworks such as the value-belief-norm theory and CN paradigm. Moreover, the results contextualize behavioral theory within a non-Western, collectivist cultural setting, highlighting the powerful role of social norms and collective moral responsibility in environmental decision-making. Practically, the findings point to several directions for promoting sustainability in higher education. Universities can enhance students’ pro-environmental engagement by reinforcing social norms that support sustainability, fostering emotional and moral attachment to nature through experiential learning, and providing structural opportunities for environmentally responsible action. Educational programs that integrate cognitive learning with effective and ethical reflection may thus be more effective in cultivating long-term ecological responsibility. Overall, this study contributes new knowledge by revealing that in collectivist educational settings, PEB is shaped less by individual capability and more by social legitimacy and morally grounded attitudes, thereby refining how TPB–VBN integrations should be interpreted and applied across cultural contexts.

## Data Availability

The original contributions presented in the study are included in the article/supplementary material, further inquiries can be directed to the corresponding author.
